# A phased SNP-based classification of sickle cell anemia *HBB* haplotypes

**DOI:** 10.1186/s12864-017-4013-y

**Published:** 2017-08-11

**Authors:** Elmutaz M. Shaikho, John J. Farrell, Abdulrahman Alsultan, Hatem Qutub, Amein K. Al-Ali, Maria Stella Figueiredo, David H.K. Chui, Lindsay A. Farrer, George J. Murphy, Gustavo Mostoslavsky, Paola Sebastiani, Martin H. Steinberg

**Affiliations:** 1 Department of Medicine, Boston University School of Medicine, 72 E. Concord St, Boston, MA 02118 USA; 2 Bioinformatics Program, Boston University, Boston, MA 02215 USA; 3 Sickle Cell Disease Research Center and Department of Pediatrics, College of Medicine, King Saud University, Riyadh, Saudi Arabia; 4 Al-Omran Scientific Chair, King Faisal University, Al-Ahssa and Institute for Research & Medical Consultation, Imam Abdulrahman bin Faisal University, Dammam, Saudi Arabia; 5 Hematology and Blood Transfusion Division, Escola Paulista de Medicina, Sáo Paulo, Brazil; 6 Department of Biostatistics, Boston University School of Public Health, Boston, MA 02118 USA

**Keywords:** SNPs, Sickle cell, Haplotype classification

## Abstract

**Background:**

Sickle cell anemia causes severe complications and premature death. Five common β-globin gene cluster haplotypes are each associated with characteristic fetal hemoglobin (HbF) levels. As HbF is the major modulator of disease severity, classifying patients according to haplotype is useful. The first method of haplotype classification used restriction fragment length polymorphisms (RFLPs) to detect single nucleotide polymorphisms (SNPs) in the β-globin gene cluster. This is labor intensive, and error prone.

**Methods:**

We used genome-wide SNP data imputed to the 1000 Genomes reference panel to obtain phased data distinguishing parental alleles.

**Results:**

We successfully haplotyped 813 sickle cell anemia patients previously classified by RFLPs with a concordance >98%. Four SNPs (rs3834466, rs28440105, rs10128556, and rs968857) marking four different restriction enzyme sites unequivocally defined most haplotypes. We were able to assign a haplotype to 86% of samples that were either partially or misclassified using RFLPs.

**Conclusion:**

Phased data using only four SNPs allowed unequivocal assignment of a haplotype that was not always possible using a larger number of RFLPs. Given the availability of genome-wide SNP data, our method is rapid and does not require high computational resources.

## Background

Sickle cell anemia affects millions worldwide and is associated with high morbidity and mortality [1]. The concentration of fetal hemoglobin (HbF) is the main pathophysiological modulator [2]. Five major haplotypes of the β-globin gene (*HBB*) cluster are associated with different levels of HbF [3, 4]. Patients with the highest HbF generally have the mildest disease [5, 6]. Therefore, classification of patients’ haplotype is useful for prognostic purposes and for studying the genetic differences that contribute to the HbF variability among these haplotypes.

**Fig. 1:**
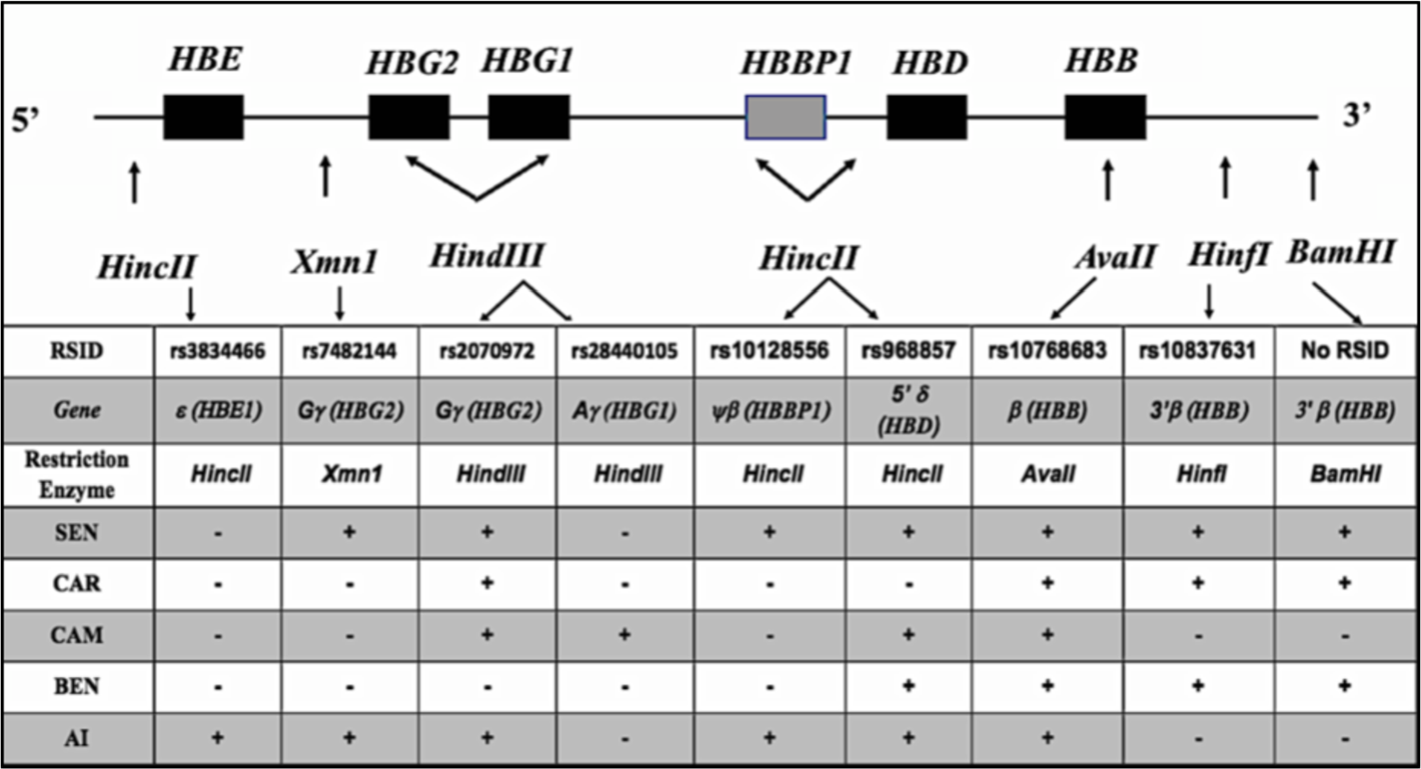
Restriction Enzyme Recognition Sites in the β-Globin Gene Cluster. RSIDs of SNPs present in restriction endonuclease sites in the β-globin-like gene cluster. (+) denotes the presence of the corresponding enzyme site while (−) denotes the absence in the five-major sickle cell haplotype (Benin (BEN), Central African Republic (CAR), Senegal (SEN), Cameroon (CAM) and Arab–Indian (AI)

Haplotypes of sickle cell anemia were first ascertained by analysis of restriction fragment length polymorphisms (RFLPs) in the *HBB* gene cluster [7]. This classification was based on detecting whether or not cleavage occurred at five to eight restriction sites when DNA was digested with restriction endonucleases, as shown in Fig. 1 [8–10]. This method is time-consuming and can lead to error [10]. Fluorescence resonance energy transfer coupled with high-resolution melting (HRM) assay is another method to classify sickle cell haplotypes, but it is also labor intensive and requires multiple laboratory assays [10]. Neither method is capable of differentiating between parental and maternal alleles in an individual so that without informative genetic data from family members, the phasing of restriction patterns is not possible, and in many cases ascertainment of a haplotype is either equivocal or impossible. We used genome-wide association study (GWAS) data imputed to a reference panel to obtain a phased output. The phased GWAS data allowed assigning SNPs to parental chromosomes, which facilitated the classification procedure using fewer SNPs.

## Methods

### Haplotype classification

GWAS data were available for patients with sickle cell anemia from the Cooperative Study of Sickle Cell Disease (CSSCD) [11]. SNP array data containing 588,451 markers were evaluated using PLINK to identify and remove SNPs with minor allele frequency (MAF) < 0.01, that violated Hardy-Weinberg Equilibrium (HWE), and had more than 0.05 missing genotype information [12]. Genotypes for a total of 560,170 SNPs were imputed using the Michigan Imputation Server [13], the 1000 Genomes Phase 3 v5 reference panel, and the Eagle phasing algorithm to obtain phased output [14, 15]. We developed a Python script based on VCF and PYSAM Python modules to read SNP information and assign the haplotype accordingly [16, 17]. Code and an example are available on GitHub (https://github.com/eshaikho/haplotypeClassifier). We used this script to classify 1394 samples that were previously classified by RFLP in the CSSCD. We selected four SNPs (rs3834466, rs28440105, rs10128556, and rs968857) which define all of the haplotypes spanning the β-globin gene cluster (Table 1).

**Table 1:** Five Major Haplotypes and Alleles of the Four SNPs Defining the Haplotype

Haplotype	rs3834466	rs28440105	rs10128556	rs968857
AI	GT	C	T	T
SEN	G	C	T	T
BEN	G	C	C	T
CAR	G	C	C	C
CAM	G	A	C	T

### Calculation of HbF average per haplotype

To check the consistency of classification and average HbF for each haplotype, we used samples with available HbF level information including 559 of the 813 samples that were successfully classified with RFLPs, 916 of the samples classified with SNP-based methodology, and 252 of samples that were either partially classified or failed classification with RFLPs. We calculated the average HbF level for each haplotype using psych R package [18], and generated a boxplot for the most common haplotypes (Benin homozygotes [BEN/BEN], Benin/Central African Republic compound heterozygotes [BEN/CAR], Benin/Senegal compound heterozygotes [BEN/SEN], Benin/Cameroon compound heterozygotes [BEN/CAM], Central African Republic homozygotes [CAR/CAR]) in this cohort to show the consistency of HbF levels across the three groups (five RFLP classification, SNP-based classification, and the group that failed five RFLP classification but were able to be classified with the SNP-based method).

### Classification of haplotypes in Saudi sickle cell anemia patients and in a library of sickle cell anemia induced pluripotent stem cells (iPSCs)

Since CSSCD patients are mostly African American, we tested our method using data obtained from sickle cell anemia patients from the Eastern and Southwestern Provinces of Saudi Arabia. Eastern Province patients tend to have the autochthonous Arab Indian (AI) haplotype as the major haplotype, while Southwestern Province patients mostly have the BEN haplotype that was introduced from Africa. The HbF levels in Saudi Benin patients is twice as high as African American patients with this haplotype [2, 19]. To further test our method on a mixed population of diverse ethnicity we reclassified haplotypes originally ascertained using RFLPs in a library of sickle cell anemia-derived iPSCs [20].

## Results

### Haplotype classification

Of 371 CSSCD patients classified as BEN/BEN using five RFLPs, we achieved a concordance of 98% (367/371) using four phased SNPs. We achieved >99% concordance for patients classified as BEN/CAR using RFLPs. For BEN/SEN, BEN/CAM, CAR/CAR, CAR/SEN, CAR/CAM, CAR/AI, SEN/SEN, SEN/CAM, SEN/AI, and CAM/CAM haplotypes our concordance with the RFLP method was 100% although the numbers of patients in each category was smaller. Two patients classified originally as BEN/AI failed reclassification (Table 2). Discordance between our method and the five RFLP method occurred in only eight of 813 patients providing an overall concordance rate > 99%. Two patients classified as BEN/AI with RFLP were reclassified as UNKNOWN/SEN. Four patients classified as BEN/BEN were reclassified as UNKNOWN/BEN, CAR/CAR, CAM/BEN, and SEN/BEN. The last two patients were classified as CAR/SEN and SEN/BEN with our methods instead of BEN/CAR according to RFLP analysis. Importantly, we were able to assign a haplotype to 86% (343/ 395) of samples from the CSSCD that failed classification using RFLPs.

**Table 2:** Comparison of Haplotype Classification Methods for 813 Sickle Cell Anemia Patients from the CSSCD

Haplotype	5 RFLPs	4 SNPs	Concordance
BEN/BEN	371	367	0.989218329
BEN/CAR	226	224	0.991150442
BEN/SEN	91	91	1
BEN/CAM	41	41	1
CAR/CAR	31	31	1
CAR/SEN	17	17	1
CAR/CAM	14	14	1
SEN/CAM	9	9	1
SEN/SEN	8	8	1
BEN/AI	2	0	0
CAR/AI	1	1	1
SEN/AI	1	1	1
CAM/CAM	1	1	1

Five RFLP represents haplotypes previously assigned by RFLPs using information from 5 restriction sites based on Southern blotting. 4 SNPs represents the number of haplotypes assigned using the phased SNP-based classification

### Calculation of HbF average per haplotype

**Fig. 2:**
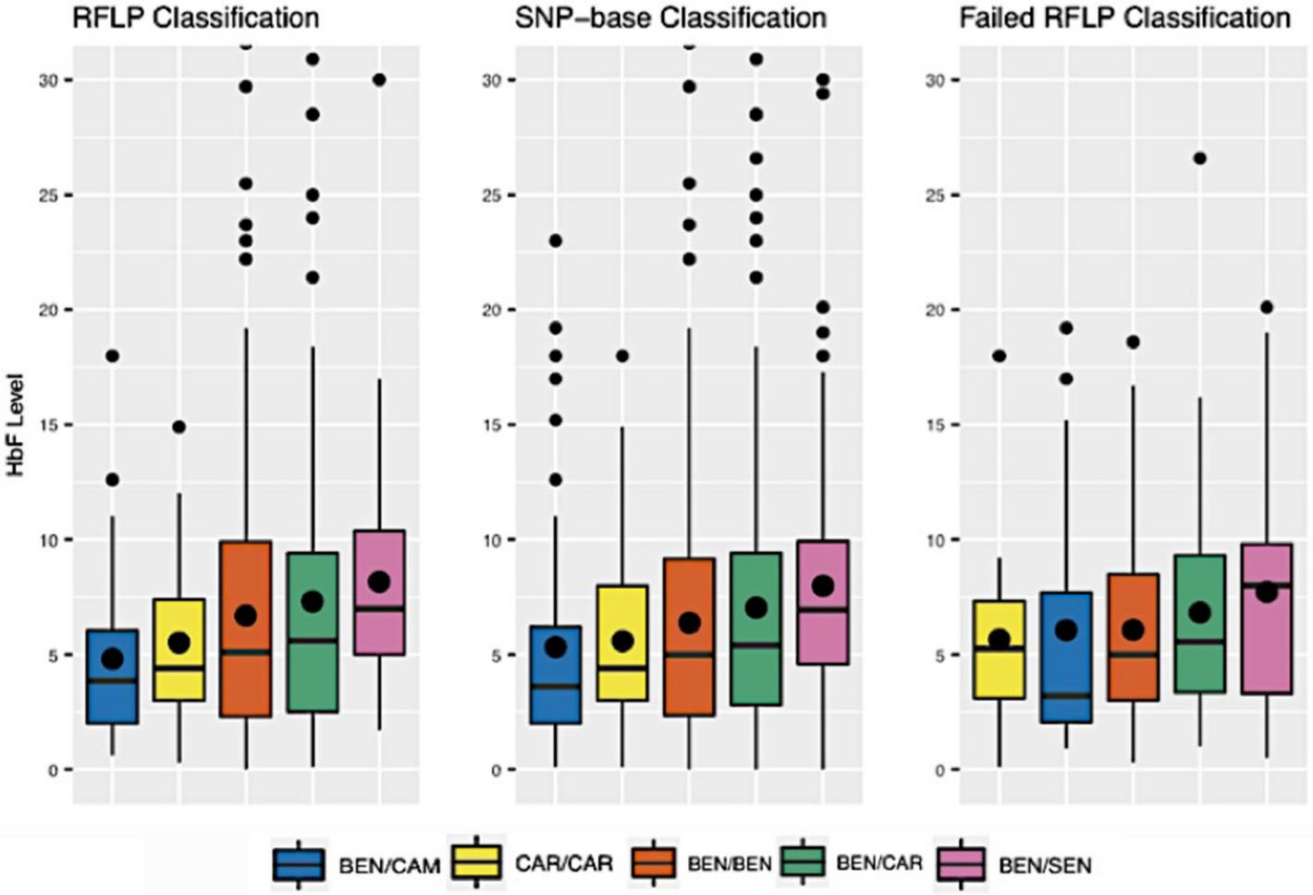
Boxplots of the Common Haplotypes in the CSSCD. Shown are the HbF levels according to haplotype defined by RFLPs and SNP-based methodology. The third panel shows data from patients not classified by RFLP but successfully classified using the SNP-based method. The *black dots* in the middle of the boxplots represent mean HbF level, while the *black horizontal lines* represent the median HbF level

The average haplotype HbF level of patients classified with our method is consistent with average HbF in haplotypes reported in literature based on RFLPs (Table 3) [5, 6]. The average haplotype HbF for samples unclassifiable using RFLPs, but classified using phased SNP data matched the average HbF for each known haplotype (Table 3). Boxplots of the most common five haplotypes in the CSSCD cohort show the consistency of HbF levels across the three classification groups (Fig. 2 **)**.

**Table 3:** Mean HbF Levels Among Haplotypes

^a^CSSCD									
	*RFLP classification*			*SNP-based classification*			*Failed RFLP classification*		
Haplotype	n	mean	sd	n	mean	sd	n	mean	sd
BEN/BEN	253	6.69	5.58	379	6.39	5.14	89	6.07	4.32
BEN/CAR	157	7.32	7.63	261	7.04	6.77	74	6.83	5.37
BEN/SEN	62	8.18	4.75	116	7.99	5.32	33	7.72	5.50
BEN/CAM	32	4.81	3.89	53	5.33	5.19	15	6.06	6.18
CAR/CAR	21	5.52	3.71	41	5.59	3.90	16	5.66	4.47
CAR/SEN	11	9.80	3.48	24	8.91	7.59	7	11.24	13.14
CAR/CAM	9	3.80	2.51	18	3.96	3.24	8	4.41	4.16
SEN/SEN	7	9.24	5.30	13	8.74	4.35	5	7.58	3.35
CAM/SEN	3	7.43	6.23	9	8.61	6.02	5	9.24	7.17
BEN/AI	2	4.20	5.37	-	-	-	-	-	-
CAM/CAM	1	7.00	-	1	7.00	-	-	-	-
CAR/AI	1	16.10	-	1	16.10	-	-	-	-
^b^SW Saudi									
	*RFLP classification*			*SNP-based classification*			*Failed RFLP classification*		
Haplotype	n	mean	sd	n	mean	sd	n	mean	sd
BEN/BEN	39	11.22	5.32	48	10.28	4.19	-	-	-
CAR/CAR	11	9.35	5.07	-	-	-	-	-	-
BEN/SEN	2	8.65	0.64	-	-	-	-	-	-
BEN/CAR	1	6.50	-	1	3.10	-	-	-	-
SEN/SEN	1	20.40	-	-	-	-	-	-	-
UNK	1	12.60	-	-	-	-	-	-	-
AI/AI	-	-	-	1	20.40	-	-	-	-
BEN/CAM	-	-	-	2	10.15	9.69	-	-	-
BEN/UNK	-	-	-	3	20.20	8.64	-	-	-
^c^E Saudi									
	*RFLP classification*			*SNP-based classification*			*Failed RFLP classification*		
Haplotype	n	mean	sd	n	mean	sd	n	mean	sd
AI/AI	30	18.03	5.39	30	18.03	5.39	-	-	-

Included are 559 patients with HbF phenotypes classified with RFLP method, 916 patients with HbF phenotypes classified with SNP-based method, and 252 samples that failed RFLP classification but were classifiable with SNP-based method. (n) is the number of patients in the corresponding haplotype class, (mean) is the mean of HbF per haplotype, (sd) is the standard deviation. ^a^CSSCD are African Americans; ^b^SW Saudi is Saudi patients from Southwestern Province; ^c^E Saudi are Saudi patients from the Eastern Province. UNK-unknown

### Classification of haplotype in Saudi sickle cell anemia and sickle iPSCs

Haplotypes among 55 Southwestern Province patients classified using the RFLP method included 39 BEN/BEN, 11 CAR/CAR, 2 BEN/SEN, 1 BEN/CAR, 1 SEN/SEN and one unknown. The distribution of haplotypes for these subjects derived using the SNP method was 48 BEN/BEN, 3 BEN/UNKNOWN, 2 BEN/CAM, and one AI/AI (Table 3). The concordance between RFLP and SNP-based classification was 67%. For the 30 Eastern Province patients, we had 100% concordance since all patients reclassified as AI/AI (Table 3).

In a library of sickle cell anemia iPSCs there was high concordance between the two methods of haplotype ascertainment. The only discordance was in two patients classified originally as BEN/BEN that according to SNP-based reclassification were CAM/BEN and CAR/SEN (Table 4). Importantly, we were able to assign a haplotype to 15 of 17 iPSC samples that were classified as either atypical or were indeterminate using RFLPs.

**Table 4:** Sickle Anemia-Specific iPSC Library

Name of Line	Gender	Nationality of Origin	Age	Haplotype	4SNPs
SA108	male	Saudi Arabia	9	AI/AI	AI/AI
SA50-1	female	Saudi Arabia	NA	AI/AI	AI/AI
SA106-1	female	Saudi Arabia	NA	AI/AI	AI/AI
SA170-1	male	Saudi Arabia	3	AI/AI	AI/AI
SS2-1	female	US	32	UNK/UNK	BEN/SEN
SS2-1GAG (CRISPR corrected)	female	US	32	UNK/UNK	BEN/SEN
SS12-1	female	US	27	UNK/UNK	BEN/CAR
SS18-1	female	US	23	UNK/UNK	UNK/UNK
SS28-1	female	US	25	UNK/UNK	BEN/BEN
SS36	male	US	38	UNK/UNK	BEN/CAR
SS41-1	male	US	21	UNK/UNK	CAR/CAR
SS45-1	female	US	37	UNK/UNK	BEN/BEN
SS47-1	female	US	42	UNK/UNK	BEN/CAR
SS48-1	male	US	30	UNK/UNK	CAR/CAR
SA5-1	female	Saudi Arabia	9	UNK/UNK	BEN/BEN
SA53-1	male	Saudi Arabia	14	UNK/UNK	BEN/CAR
SA208	male	Saudi Arabia	7	UNK/UNK	UNK/BEN
SA138-1	male	Saudi Arabia	16	UNK/UNK	AI/AI
BR-SP-21-1	female	Brazil	20	UNK/UNK	BEN/CAR
BR-SP-37-1	female	Brazil	20	UNK/UNK	CAR/CAM
BR-SP-45-1	female	Brazil	20	UNK/UNK	CAR/CAR
SS24-1	male	US	24	CAR/CAR	CAR/CAR
SS25-1	female	US	22	CAR/CAR	CAR/CAR
BR-SP-3-1	female	Brazil	34	CAR/CAR	CAR/CAR
BR-SP-23-1	female	Brazil	23	CAR/CAR	CAR/CAR
BR-SP-25-1	male	Brazil	34	CAR/CAR	CAR/CAR
BR-SP-41-1	male	Brazil	22	CAR/CAR	CAR/CAR
BR-SP-43-1	male	Brazil	21	CAR/CAR	CAR/CAR
SS9-1	female	US	29	BEN/CAR	BEN/CAR
SS13-1	female	US	25	BEN/CAR	BEN/CAR
SS15-1	female	US	28	BEN/CAR	BEN/CAR
SS35	male	US	50	BEN/CAR	BEN/CAR
BR-SP-29-1	male	Brazil	20	BEN/CAR	BEN/CAR
BR-SP-33-1	female	Brazil	53	BEN/CAR	BEN/CAR
BR-SP-39-1	male	Brazil	22	BEN/CAR	BEN/CAR
SS5-1	male	US	32	BEN/BEN	BEN/BEN
SS14-1	female	US	39	BEN/BEN	BEN/BEN
SS16-1	female	US	36	BEN/BEN	BEN/BEN
SS19-1	male	US	30	BEN/BEN	BEN/BEN
SS29-1	female	US	32	BEN/BEN	BEN/BEN
SS32	female	US	33	BEN/BEN	BEN/BEN
SS37	female	US	37	BEN/BEN	BEN/BEN
SS38	male	US	26	BEN/BEN	BEN/BEN
SS44-1	female	US	23	BEN/BEN	BEN/CAM
SS49-1	male	US	31	BEN/BEN	CAR/SEN
SA36	female	Saudi Arabia	26	BEN/BEN	BEN/BEN
SA40-1	male	Saudi Arabia	20	BEN/BEN	BEN/BEN
SA64	male	Saudi Arabia	14	BEN/BEN	BEN/BEN
SA82-2	male	Saudi Arabia	24	BEN/BEN	BEN/BEN
SA209-1	male	Saudi Arabia	12	BEN/BEN	BEN/BEN
SA210-1	male	Saudi Arabia	9	BEN/BEN	BEN/BEN
BR-SP-31-1	male	Brazil	35	BEN/BEN	BEN/BEN
SS4-1	male	US	30	BEN/SEN	BEN/SEN
SS8-2	female	US	31	SEN/SEN	SEN/SEN
SS43-2	female	US	32	SEN/SEN	SEN/SEN

Haplotype denotes the haplotype classification and 4 SNPs is SNP-based reclassification. The table is modified from [20]

## Discussion

In adults, homozygotes for the BEN, CAR, and CAM haplotypes were associated with HbF of 5-7% of total hemoglobin; SEN and AI haplotypes had HbF levels of about 10% and 20%, respectively. Using GWAS data we were able to classify with high accuracy and time efficiency the haplotype of sickle cell anemia patients using four SNPs. The primary feature of our classification method is a phasing step after genotype imputation where SNP alleles are assigned to parental chromosomes, and the haplotype of each chromosome is assigned independently. This method was superior to ascertaining haplotype by RFLP using unphased SNPs at five sites and was successfully applied in a few seconds on a personal computer.

Haplotyping errors can occur using the SNP-based method because of the SNP genotyping platform, imputation errors, and ambiguities arising from phasing algorithms. Nevertheless, in African-origin patient samples, we were able to achieve a concordance of 99% percent (805/813) between 4-SNP haplotypes derived from a phasing algorithm using GWAS data with 5-SNP haplotypes determined using restriction analysis. Haplotype assignment in a sickle cell anemia iPSC library also showed high concordance and demonstrated the efficiency of SNP-based method to classify samples that failed RFLP classification. The discordance between SNP-based and RFLP ascertainment most likely resulted from errors in the RFLP classification that is sensitive to the presence of other SNPs in the restriction sites and the vagaries of restriction enzyme analysis and Southern blotting that was used for haplotype analysis in the CSSCD.

In 30 Saudi East patients where the AI haplotype was ascertained by genotyping rs7482144 (Xmn1 5′ to *HBG2*), rs3834466 (Hinc2 5′ to *HBE1*), and rs549964658 (5′ to *HBD*) we had 100% concordance.

The major discordance between RFLP and SNP-based analysis for classification of Saudi Southwestern patients occurred among 11 subjects classified as CAR/CAR. Eight of these 11 were reclassified as BEN/BEN and three as BEN heterozygotes. The only difference between CAR and BEN haplotypes is the SNP rs968857 at the *HincII* site 5′ of *HBD* (Fig. 1 **,** Table 2). It is most likely that this discrepancy was a result of an error in RFLP analysis. If the discordance was due to imputation quality, the error rate would probably match the imputation error. There is 100% discordance at this *HincII* site while the imputation quality score of rs968857 is R^2^ = 0.99. One patient with HbF of 20.4% originally as SEN/SEN by RFLP was reclassified as AI/AI.

To investigate the discordance in Southwestern Province patients, we examined the genotype data of the *HBB* gene cluster downstream of *OR51V1* (5′ olfactory receptor gene cluster) and upstream of *OR51B4* (3′ olfactory gene cluster) in both patient groups. The SNP genotypes of the 11 CAR/CAR that we reclassified as BEN/BEN or BEN heterozygous had the same SNP genotype of BEN/BEN patients that were classified as such with both methods. The genotype data, average HbF, and the imputation quality score of rs968857 suggest that the high discordance in Southwestern Saudi patients is due to RFLP errors.

A limitation of our method is the dependency on the availability of GWAS data for many SNPs in the β-globin gene region. However, many large patient cohorts have been genotyped using genome-wide SNP arrays. In these patients, haplotype information might be useful as a covariate in a genetic risk analysis. RFLP analysis might be suitable for a small number of patients but requires optimization of all of the individual assays. The main advantage of our haplotype determination method is the rapid classification and high accuracy. This method can also be used for whole genome sequence data classification after SNP calling and phasing. Moreover, it is not sensitive to SNPs that alter the restriction enzyme recognition sequence that can lead to error using RFLPs.

## Conclusion

Phased data using only four SNPs allowed unequivocal assignment of a β-globin gene cluster haplotype that was not always possible using a larger number of RFLPs, and was also more accurate. With the availability of genome-wide SNP data our method is rapid and does not require high computational resources.
